# Nervous Mimicry of Organic Diseases

**Published:** 1874-06

**Authors:** James Paget


*THE LONDON LANCET.—January*, 1874:

Nervous Mimicry of Organic Diseases.—*By Sir James Paget.*—Cases of this kind are commonly included under the name Hysteria; but in many of them none of the distinctive signs of hysteria are ever observed, and from all of them it is desirable that this name should be abolished. For it is absurdly derived, and, being often used as a term of reproach, is worse than absurd. To call a patient hysterical is taken by many people as meaning that she is silly, or shamming, or could get well if she pleased; and no doubt there are patients of whom some of these things may be fairly said; but in many more, hysteria, especially in the form of an unwilling imitation of organic disease, is a serious affection, making life useless and unhappy and not rarely shortening it.

But the characters of nervous mimicry are distinct enough to make a separate group with another name. In English we may speak of nervous mimicry; in untranslated Greek, of neuromimesis. To patients and their friends the maladies may be said to be due to extreme nervous sensibility; or, if they also prefer Greek, we may call them hyperneurotic: anything but hysterical.

Now, there is scarcely a local organic disease of invisible structures which may not be mimicked by nervous disorder. You hear of hysteric cough and hysteric dyspepsia and paralysis, of hysteric joints and spines.

The means for diagnosis are to be sought—(1) in what may be regarded as the predisposition—the general condition of the nervous system on which, as on a predisposing constitution, the nervous mimicry of disease is founded; (2) in the events by which, as by exciting causes, the mimicry may be evoked or localised; (3) in the local symptoms of each case.

It is seldom that patients with well-marked nervous mimicries have ordinary minds—such minds as we may think average, level, and evenly balanced. You may, indeed, find among them some common-place people, with dull, low-level minds; but, in the majority, there is something notable, good or bad, higher or lower, than the average—something outstanding or sunken.

Nothing can be more mischievous than a belief that mimicry of organic disease is to be found only or chiefly in the silly, selfish girls among whom it is commonly supposed that hysteria is rife or an almost natural state. It would be safe for you to believe that you are likely to meet with it among the very good, the very wise, and the most accomplished among women. But it will be safest if you believe only that, in any case of doubt whether a local disease be organic or nervous, it adds something to the probability of its being nervous if the patient has a very unusual mental character, especially if it be unusual in the predominance of its emotional part.

Sometimes there is a general feebleness of will; the patients can do nothing for themselves; can trust themselves in nothing; but commit themselves to some one with a stronger will, and an appearance, if not a reality, of more knowledge. Hence, among these patients are the most numerous subjects of mesmerism, and the other supposed forces of which the chief evidence is the power of a strong will over a weak one.

A girl who has will enough in other things to rule the house has yet not enough in regard to her limbs to walk a step with them, though they are as muscular as ever in her life. In will these patients are as those who are color-blind. They say "I cannot;" it looks like "I will not;" but it is "I cannot will."

But to regard all mimicries of organic diseases as essentially mental errors would be bad pathology and worse practice.

Some mimicries are essentially mental. But in some it is hard to discern any mental influence.

Some are found in common-place, ignorant, and slow-minded people who never saw or heard of the disease imitated in them. Some occur in children who could neither imagine nor act what they tell and show.

And whatever may be ascribed to be mental influence, it can produce mimicry of organic disease in only certain persons whose nervous organs seem wholly prone to this manner of disorder, and whose spinal and ganglionic systems must be deemed erroneous, as well as, or more than, their brains. For nervous mimicry is not very frequent among the evidently insane, and among the sane there are many who cannot bring about a mimicry of disease by any effort of imagination or direction of the mind.

Often the inconsistency of a quick pulse with only natural or slow breathing and a low temperature may nearly suffice to tell that some very painful disease of long standing is only "nervous."

The temperature of a patient in whom you are doubting between real and mimic disease should always be observed.

Speaking generally, it is not affected in any degree proportionate to the signs which may seem like those of acute disease.

And this inconsistency may settle your doubts. But if the temperature be variable, or often high, you must be cautious.

And it may often be observed that, though with little or no organic disease, a nervous patient's temperature may be normal, or not above 101 deg., yet with a moderate addition of acute disease the temperature may rise much higher than it would in any one with a healthy nervous system. I perforated an abscess in a very hysterical young lady's tibia; a few days afterwards the escape of pus was casually hindered, and in that evening her temperature rose to 105 deg. In the next evening it was 104·1 deg. In the following morning it was 100·5 deg.; in the evening 101·5 deg.; and then it fell nearly to normal. And this had happened without any material pain or inflammation; and, even when the temperature was 105 deg. she was cheerful, and with a pulse about 100. Her respiration was natural.

These facts may be enough for caution against over-reliance on any one sign of disease in patients of nervous constitution, even though it be the measurable temperature. Prudently estimated, it is of the highest value, even in nervous patients; over-estimated, it is more in them than in any others.

In the defective ovarian and uterine functions of certain patients some see the center and chief substance of the whole disease; a very mischievous fallacy. Of course the sexual organs appear generally in fault to those who are rarely consulted for the diseases of any other part; but in general practice they are, in a large majority of cases, as healthy as any other parts are, or not more disturbed. The close and multiform relations of the sexual organs with the mind, and with all parts of the nervous system, are enough to make the disorders of these organs dominant in a disorderly nervous constitution; but their relation to "hysteria" or to "neuromimesis," though more intense, is only the same in kind as that of an injured joint or an irritable stomach.

